# Considerations and Challenges for Acute Inhalation Toxicity Testing and Classification of Zinc Sulphide Under REACH

**DOI:** 10.3390/toxics13010027

**Published:** 2024-12-31

**Authors:** Gustav Gerd Bruer, Noömi Lombaert, Arne Burzlaff, Christine Spirlet, Daria Gödecke, Mehmet Ramazanoglu, Otto Creutzenberg

**Affiliations:** 1 Fraunhofer Institute for Toxicology and Experimental Medicine (ITEM), Nikolai-Fuchs-Str. 1, 30625 Hannover, Germany; 2 International Zinc Association, 168 Avenue de Tervueren, 1150 Brussels, Belgium; 3 EBRC Consulting GmbH, Kirchhorster Str. 27, 30659 Hannover, Germany

**Keywords:** zinc sulphide, inhalation toxicology, acute toxicity, REACH, rats

## Abstract

Zinc sulphide is a widely used inorganic powder, and its production has reached quantities greater than 1000 t/year. Therefore, in accordance with OECD guideline 436, an acute inhalation test was implemented to provide more accurate data. This study is crucial for ensuring the safety of workers exposed to zinc sulphide dust and complying with regulatory requirements for REACH. Due to particle-specific properties, the maximum attainable concentration of zinc sulphide for an inhalation study was not certain. Two dry dispersion systems were used to aerosolize the zinc sulphide powder, and the generated aerosol was supplied to a nose-only inhalation exposure system. The results showed a maximum attainable concentration of 0.82 mg/L at an MMAD of 1.5 µm over a 4 h exposure. In the inhalation study, all six rats showed no specific symptoms and good health status and survived a post-exposure observation period of up to 14 days. From the results observed, the status of Not classified was derived according to CLP. Based on the experimental results, an LC50 was not determined but is considered to be higher than 0.82 mg/L (the maximum achievable aerosol concentration). These findings highlight the importance of documenting efforts to achieve aerosol conditions when concentrations required by OECD test guidelines cannot be reached.

## 1. Introduction

Zinc sulphide (ZnS; CAS 1314-98-3) is the most prevalent form of zinc found in nature. The naturally occurring mineral of zinc sulphide is sphalerite [1]. Zinc sulphide is a widely used inorganic powder, mainly as a phosphorescent material in watches and TV screens, as an infrared optical material, as a white pigment, and in dental materials [2]. The electronics and semiconductor industry is a significant driver of the zinc sulphide market. Zinc sulphide production has reached quantities greater than 1000 t/year, and Zinc Sulphide Registration Dossier from EU REACH (Registration, Evaluation, Authorization and Restriction of Chemicals) has reported a total tonnage band ≥ 100,000 to <1,000,000 tons (manufactured or imported). For the acute hazard identification of chemicals manufactured or imported at quantities above 10 tons per year, REACH requires the two most appropriate routes of administration (REACH Annex VIII, [3]) to be tested. Acute toxicity testing via the oral route is mandatory, whereas selection of the dermal or inhalation route requires expert judgment. The inhalation route is selected if it is anticipated to be the most relevant route of human exposure (according to REACH Annex VIII, Section 8.5, Column 2) [3].

REACH Annex VIII also states that “before new tests are carried out to determine the properties listed in this Annex, all available in vitro data, in vivo data, historical human data, data from valid (Q)SARs and data from structurally related substances (read-across approach) shall be assessed”. The ‘3Rs’ principle of Replacement, Reduction, and Refinement of the use of animals in scientific research—proposed by Russell and Burch in “The Principles of Humane Experimental Technique” [4]—is a key element of the REACH legislative text. Testing on animals should only be undertaken as a last resort—when there is no other scientifically reliable way to examine the impact of chemicals on humans or the environment.

Nowadays, there are many more non-animal approaches available, such as in silico tools, in vitro assays, omics, defined approaches, next-generation risk assessment, as well as initiatives dedicated to the development of non-animal approaches for every endpoint in REACH, including complex endpoints [5,6,7]. However, currently, there is no in vitro model that has been accepted by regulatory agencies as a stand-alone replacement for animal tests in acute inhalation toxicity studies, and the issues associated with interspecies differences remain unsolved. Although in some cases, in vitro alternative tests are at an advanced stage of development (e.g., the EpiAirway^TM^ model), to date, all in vitro alternative models for inhalation toxicity studies still fall in the category of “non-guideline methods” [8]. However, for regulatory purposes, acute toxicity data are required for the classification and labeling of chemicals according to their intrinsic toxicity. Therefore, REACH defines the information requirements, which mainly depend on the volume of the substance produced or imported and the intrinsic hazardousness of the substance. Previous assessments for fulfilling the data requirement of acute toxicity testing for zinc sulphide used a read-across approach from other inorganic slightly soluble zinc substances. This read-across strategy was based on the bioavailability of the zinc ion.

Zinc sulphide belongs to the category of inorganic zinc substances. The inhalation read-across for this category uses information such as (i) solubility in relevant biological media, (ii) effects towards mucous membranes, and (iii) effects following short-term inhalation exposure to read-across from source to target substances. However, for zinc sulphide, based on the available evidence, it is hypothesized that a complete absence of effects can be expected in an acute inhalation toxicity study.

Since no publicly available specific acute inhalation toxicity test data were available for zinc sulphide, an acute inhalation test on the latter, in accordance with OECD guideline 436 [9], was performed to verify the read-across hypothesis, i.e., zinc sulphide being void of any adverse effects after acute inhalation exposure. Based on the abstract by Bruer et al. (2024) [10], this paper aims to report on the acute inhalation toxicity testing of zinc sulphide, along with its challenges and its outcome in terms of classification purposes.

## 2. Materials and Methods

### 2.1. Animal-Free Experiments—Maximum Attainable Aerosol Concentration

Animal-free measurements to determine the maximum attainable chamber atmosphere concentration of zinc sulphide were performed by Fraunhofer ITEM (Hannover, Germany). These animal-free tests were conducted using the same inhalation chambers used for the in vivo acute inhalation toxicity testing but with two different dispersion systems. The first system was a commercial dispersing system from TOPAS GmbH (TOPAS GmbH, Dresden, Germany, SAG 410/L), and the second system was a dispersion system, which was developed by Fraunhofer ITEM (Trido + Wischonsky-Nozzle). The most effective aerosol generation was achieved using the Trido generator. It is capable of dispersing a powder constantly through a segmented conveyor. The TOPAS generator was not suitable for the dispersion of zinc sulphide because of the clogging of the nozzle and the conveyor belt after 5 min. Based on these preliminary experiments, the maximum attainable chamber atmosphere concentration was determined as 0.85 mg zinc sulphide/L.

### 2.2. Acute Inhalation Toxicity Study

The acute inhalation toxicity study according to OECD guideline 436 on the test item, i.e., zinc sulphide, was performed by Fraunhofer ITEM (Hannover, Germany). The specifications of the test item are shown in Table 1.

#### 2.2.1. Animal Model

Young adult male and female Wistar rats (three per sex and test group) were purchased from Charles River Deutschland, Sulzfeld, Germany, at approximately 6–7 weeks of age. Wistar rats are commonly used in toxicity studies. Upon arrival, all animals were thoroughly examined by a veterinarian. This examination included checking the whole body, especially the skin, hair coat, eyes, ears, nose, orifices, and respiratory tract. Only animals with satisfactory general health status were included in this study. Before the treatment period, the animals were allowed to acclimate to the Fraunhofer ITEM environment for approximately two weeks, during which clinical observations were made at least once a day. Rats were accepted for this study after this period, provided they showed no signs of disease and maintained a general physical condition deemed satisfactory.

Each animal was assigned a unique four-digit identification number. The animals were housed in Makrolon^®^ type IV polycarbonate cages, with three rats of the same sex per cage, under conventional laboratory conditions. The cages and bedding material were changed weekly or as needed. Fresh tap water was provided weekly or as needed, and they were fed commercial pellet chow (Ssniff V1534). Temperature and humidity in the animal room were monitored electronically and were set to 22° ± 2 °C and 55% ± 15%, respectively. A 12 h light/dark cycle was maintained, and the air exchange rate was at least 10 times per hour.

On the scheduled sacrifice date, which was day 14 post-exposure, all rats were humanely killed by cutting the vena cava caudalis after anesthesia with a lethal overdose of pentobarbital sodium (Narcoren^®^).

#### 2.2.2. Experimental Design

The rats were exposed to the exposure atmosphere in a direct flow nose-only inhalation exposure system. The inhalation route of exposure was appropriate since one potential route of human exposure to the test item is inhalation. For 2 weeks before exposure, the rats were trained to the exposure tubes, avoiding undue stress on the animals. Animal restraining tubes are constructed in such a way that hyperthermic effects on rats cannot occur. The rats are placed around the exposure cylinder in tapered acrylic glass tubes with adjustable backstops.

To allow for adequate exposure of all relevant regions of the respiratory tract, we produced an aerosol with a mass median aerodynamic diameter (MMAD) in the range of 1–4 µm and a geometric standard deviation (GSD) in the range of 1.5 to 3.0 (according to OECD guideline 436).

#### 2.2.3. Aerosol Generation

**Figure 1:**
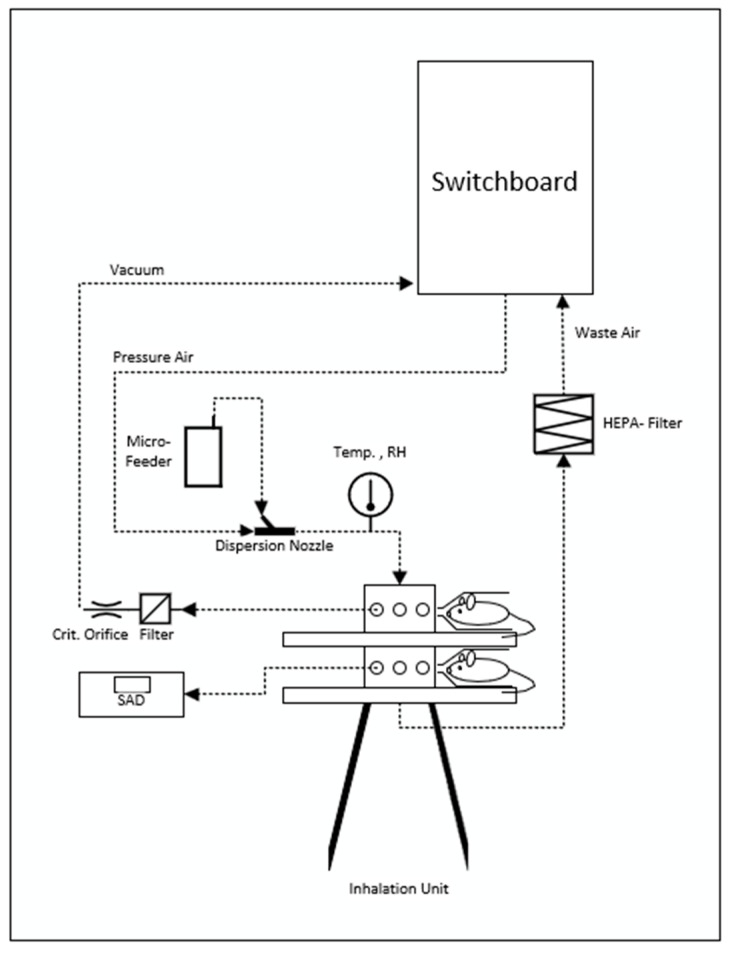
Experimental set-up of the nose-only inhalation system. The animals are placed horizontally at the ports; the port position is at the central vertical axis on the level of each of the four platforms (in the schematic, only platforms are visible as horizontal bars). The arrow indicates the air flow. SAD: Scattered light aerosol detector and RH: Relative humidity.

The aerosol was supplied to the rats by a flow-past nose-only-inhalation exposure system, which has been used for previous inhalation studies at Fraunhofer ITEM (Figure 1) and is recommended by OECD guideline 436 as the preferred procedure. The animal’s snout protrudes in the anterior end of the tube, which is connected to the exposure cylinder through a push fit. The aerosol enters the nasal region of the animal through a small tube. The exposure cylinder was operated at a slightly positive pressure with respect to the surrounding air. This ensured that a continuous airflow was passing through the animal’s breathing zone. In this system, the aerosol was supplied to each rat individually, and the exhaled air was immediately removed. Therefore, oxygen supply was always sufficient, and measurement of the oxygen concentration was unnecessary. Previous measurements with various substances have confirmed that there are no differences in concentrations among the different outlets. The exposure unit is capable of housing up to 16 animals on one level (in this study, three animals/sex were exposed).

The airflow or aerosol flow to each rat was approximately 1 L/min, which was assumed to be laminar. The total flow rate was approximately 40 L/min; the total volume of the inhalation system ensured that the intended concentration of the test item was reached shortly after the start of exposure (50%–value of concentration after approximately three minutes).

**Figure 2:**
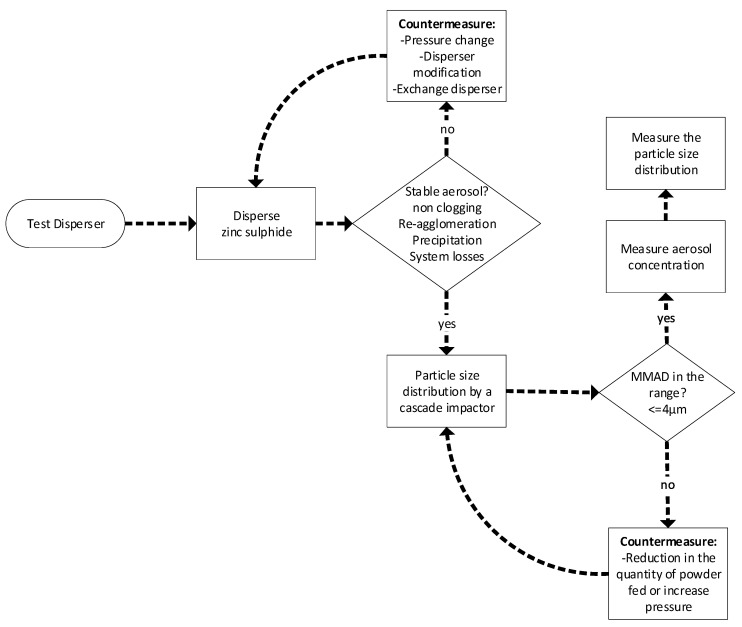
Flowchart for a series of tests to make zinc sulphide airborne according to the requirements of OECD guideline 436.

The test item aerosol was generated by dispersing the dry powder. Dispersion was achieved by a feeding system and a high-pressure and high-velocity pressurized air dispersion nozzle developed by Fraunhofer ITEM. It was selected based on the pre-tests shown in the flowchart in Figure 2 to characterize and optimize the physical properties of the aerosol and to investigate aerosol re-agglomeration and precipitation generating mass loss at the unit outlet. The feasible limit for a non-clogging exposure system was determined using this equipment (Bruer and Ramazanoglu, 2022) [11]. The disperser was fed with the test substance under computerized control, i.e., with a feedback loop to the actual aerosol concentrations measured by an aerosol photometer developed at Fraunhofer ITEM.

The signal of an aerosol photometer was used to control the feed rate of the dispersion system to keep the aerosol concentration in the inhalation unit as constant as possible. To adjust the photometer, the aerosol concentration was determined gravimetrically using filter samples (four times per four hours of inhalation). Actual test item concentrations and the mass median aerodynamic diameters were determined in the breathing zone of the animals.

Airflow, temperature, and relative humidity were measured continuously and recorded as 10 min means. The limits were set to 22 ± 2 °C for temperature and 55% ± 15% for relative humidity.

Filter samples of the aerosol were taken to control the aerosol concentration. These samples were collected at a port of the nose-only exposure unit, i.e., under the same conditions the rats were inhaling the aerosol. The mass median aerodynamic diameter (MMAD) was determined twice during the exposure period by a cascade impactor.

#### 2.2.4. Limit Test

The criterion for a positive main test is the determination of an LC50 value from up to three dose groups.

Based on the recommendations of OECD guideline 436, the first test group was exposed once for 4 h to a test item concentration of 0.85 mg zinc sulphide/L (equivalent to 850 mg/m^3^), which corresponds to the maximum attainable concentration. The study design is shown in Table 2.

**Figure 3:**
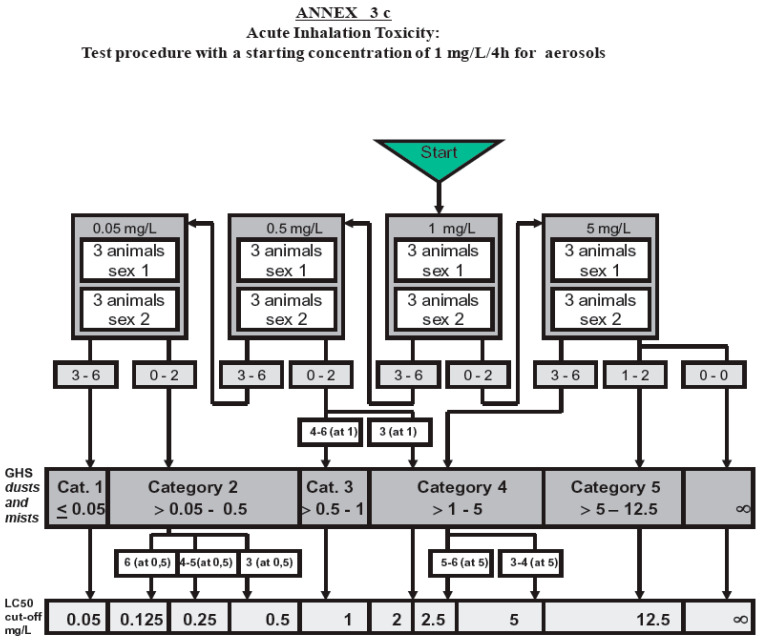
OECD TG 436 Annex 3 c [9]. For the test substance zinc sulphide, it was technically not feasible to reach the required maximum concentration of OECD guideline 436 of 5 mg/L. In this case, the authority specifies that the study should be carried out with the technically maximum attainable concentration (“the maximum attainable concentration should be tested”). This was set to 0.85 mg/L for zinc sulphide (refer to Measurement report by Fraunhofer ITEM, 7 November 2022) [11]. Because all 6 animals survived the starting concentration of 0.85 mg/L, this study was successfully completed using this concentration for testing (=limit test). Lower concentrations did not need to be tested.

Depending on the result of the first test concentration (6 animals, 3M/3F), further concentrations according to OECD guideline 436, ANNEX 3 c (Figure 3) should be tested in a maximum of two further dose groups (=full test). The doses to be tested should depend on the results of test group 1. Testing of further dose groups was not necessary in this study because the highest concentration tested was sufficient to comply with the test objective. The test objective was reached with a single concentration of 0.85 mg/L because it provided the necessary data to determine the toxicity of the test item, and additional testing at different concentrations was deemed unnecessary according to the guidelines (limit test).

It should be noted that there was a deviation from the guideline such that 0.85 mg/L, (equivalent to 850 mg/m^3^) instead of 5 mg/L (5000 mg/m^3^), was used as the potential highest dose as this was the only way to ensure the required MMAD (=1–4 µm).

#### 2.2.5. Parameters

##### Clinical Observations

On the day of treatment, all animals were frequently observed for clinical symptoms before, during, and after exposure. In the following two weeks, all animals were clinically observed in their cages once a day. Once a week, they were inspected outside their home cages and carefully examined for abnormalities in their general condition, focusing on the following parameters: general condition, fur, grooming activity, visible mucous membranes, behavior, and locomotor activity (including lethargy, coma, convulsions, diarrhea, and salivation), central nervous symptoms, breathing pattern, reflexes (at 1 h, 24 h, and 48 h after treatment), and rectal temperature (measured once after 1 h).

##### Gross Pathology/Necropsy of Respiratory Organs

Preservation and fixation of the respiratory tract: The lung and the lower half of the trachea were weighed and stored for optional histopathology. The lung was inflated under a pressure of about 20 cm water column with formalin and fixed by immersion.

### 2.3. Dustiness Test

Zinc sulphide was also tested for dustiness according to the modified Heubach method (DIN 55992-1) [12] in a small rotating drum, and the mass median aerodynamic diameter of the airborne fraction was determined using a seven-stage cascade impactor.

Based on the dustiness results and the MMAD (GSD) of the airborne fraction, the MPPD model (version 3.04) was used to predict the fractional deposition in the respiratory tract of humans.

## 3. Results

### 3.1. Aerosol Characteristics

Airflow, temperature, and relative humidity were measured continuously and recorded as 10 min means. The limits were set to 22 °C + 3 °C for temperature and 30% to 70% for relative humidity. Animal room lighting was on a 12 h light/dark cycle controlled by an automatic timing device.

For the adjustment of the photometer, the aerosol concentration was determined gravimetrically using filter samples (four times per 4 h of inhalation) (Table 3). Additionally, the MMAD was determined twice during the exposure period using a cascade impactor.

The consolidated final mean aerosol concentration was calculated based on the continuously recorded aerosol photometer values (voltage-mV) during the 4 h exposure period and resulted in a concentration of 820.44 mg/m^3^ zinc sulphide.

The respirability of the aerosol (OECD guideline 436 recommendations allow the following ranges: MMAD = 1–4 µm; GSD = 1.5–3) was achieved: MMAD (GSD) = 1.5 µm (2.0).

### 3.2. Clinical Observations

Clinical observations showed no abnormalities. No signs of local or systemic toxicity were observed in the animals. No unscheduled deaths occurred during the study period. Consequently, an LC50 was not determined in this study.

Rectal temperatures were measured in all rats approx. 1 h after the end of exposure. They were in the range of temperatures measured in historical control animals.

Individual body weight was recorded to the nearest 0.1 g on the day of exposure, before exposure (day 0), and on days 1, 3, 7, 10, and 14 before necropsy, as well as at the time of death or euthanasia for all animals. All animals lacked significant alterations in body weight data.

### 3.3. Gross Pathology/Necropsy of Respiratory Organs

During the study period, no animal was judged to be moribund by the veterinarian or his designate. Therefore, all animals at terminal sacrifice were killed humanely by cutting the vena cava caudalis after anesthesia with an overdose of pentobarbital sodium (Narcoren^®^) and necropsied immediately. These animals were subjected to a complete necropsy, which included careful examination of the external surface of the body, all orifices, and the cranial, thoracic, and abdominal cavities and their contents. In all cases, dead animals were identified by reference to each animal’s identification number.

The abdominal cavity was opened, and the diaphragm was cut carefully allowing the lungs to collapse. The heart, esophagus, upper half of the trachea, thymus, and lung-associated lymph nodes (LALN; mediastinal and tracheobronchial) were removed from the lung convolution.

All animals were sacrificed on scheduled dates. No test item-related macroscopical findings were detected. Lung weights were in the normal range of untreated rats at the given age compared to historical control data (males: approx. 1.3 g; females: approx. 1.1 g).

### 3.4. Dustiness Test

**Figure 4:**
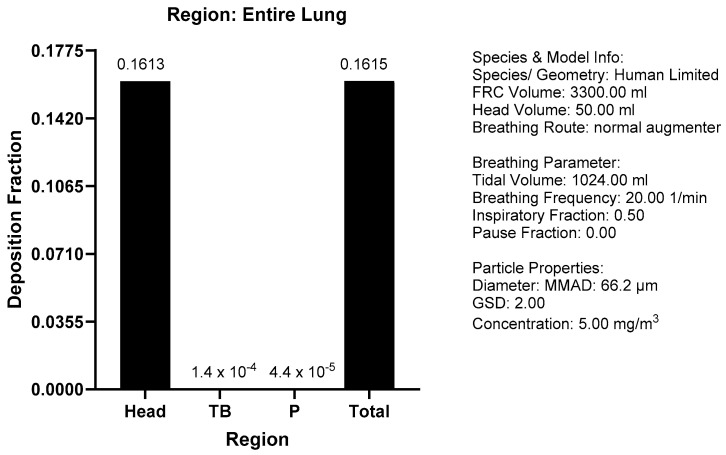
MPPD modeling [13] for deposition of ZnS in the human respiratory tract based on mean values of MMAD and GSD obtained using the modified Heubach method (calculated using MPPD V3.01, ARA 2015 and illustrated using GraphPad Prism version 10.1.2 for Windows, GraphPad Software, Boston, MA, USA).

Zinc sulphide showed low dustiness (91.28 mg/g) with the modified Heubach method [12], resulting in a MMAD (GSD) of 66.2 µm (2.0). Therefore, the deposition pattern in the human respiratory tract was predicted using the MPPD (v3.04) model, which showed that only about 0.0004% of the total handled material is predicted to be deposited in the pulmonary region. Similarly, the deposition in the head (16.13%) and tracheobronchial (0.014%) was low (Figure 4).

## 4. Discussion

As mentioned earlier, the REACH regulation requires an acute hazard identification for chemicals manufactured or imported at quantities above 10 tons per year for the two most appropriate routes of administration (REACH Annex VIII). While the oral route is mandatory, the selection of additional routes requires expert judgment.

Column 2 of the REACH Annex VIII lists specific rules according to which the required standard information may be omitted, replaced by other information, provided at a different stage, or adapted in another way (REACH Annex VIII). An acute dermal toxicity study is scientifically not necessary if the substance does not meet the criteria for classification as acute toxicity or STOT SE (specific target organ toxicity–single exposure) by the oral route and no systemic effects have been observed in in vivo studies with dermal exposure (e.g.**,** skin irritation and skin sensitization) (according to Column 2 of REACH Annex VIII). These conditions are met for zinc sulphide. For more information, please refer to the chemicals database of the European Chemicals Agency, Zinc Sulphide Registration Dossier [14]. Since currently, there is no in vitro model that can replace a regulatory-compliant acute toxicity study, an acute inhalation test on zinc sulphide was performed.

Various technical guidelines are currently provided for acute inhalation toxicity testing (OECD guidelines 403 [15], 433 [16], and 436 [9]) of chemical substances. Each is tailored to different testing needs and ethical considerations regarding animal use. Their primary goal is to determine the lethal concentration (LC50), which is the concentration that causes death in 50% of the test animals. Lethality or redemption under moribund condition is observed during a period of 14 days after exposure, and mortality, clinical signs of toxicity, and changes in body weight are monitored.

OECD guideline 403 is the traditional approach for assessing acute inhalation toxicity. Because of its detailed nature, OECD guideline 403 typically requires more animals, often using three concentration levels with at least five animals per sex per concentration. OECD guideline 433 focuses more on non-lethal toxicity, without necessarily leading to death, but it may not provide an exact lethal concentration and might result in less detailed data on the precise dose–response relationship, particularly at the higher, potentially lethal, concentrations.

In this study, OECD guideline 436 was chosen as it fulfills the aspect of animal welfare using a stepwise approach of a given dosing scheme. A dataset for classification that includes the determination of an exact LC50 can be obtained using a minimal number of animals. It is important to note that this study was conducted prior to the update of OECD guideline 403 [17]; consequently, OECD guideline 436 was selected to minimize animal use through its stepwise approach. With the revised version of TG 403, a comparable assessment might now be achievable.

Often, in particular with fluffy dust under examination, it is technically difficult to obtain a satisfying dispersion at the high aerosol concentrations requested in these acute inhalation studies. The maximum concentration tested is typically 2000 mg/m^3^; at this value, smaller airborne particles can show a strong tendency to re-agglomerate to bigger structures. In the case of zinc sulphide, it was found in technical pre-tests that the obligatory MMAD = 1–4 µm of an aerosol could only be realized at stable conditions for 4 h at ≤800 mg/m^3^.

Based on this information, the German REACH helpdesk (REACH-CLP-Biozid Helpdesk, 2021, [18]) was consulted to ensure an appropriate handling of the potential required studies. The following answer was obtained:

- “A valid justification needs to be provided demonstrating how the maximum attainable concentration was achieved, which and how many methods have been tested/ used for aerosol generation and why a higher concentration was not attainable by any of these methods”
- “In addition and if applicable, it needs to be justified why further animal testing is considered (not) appropriate”
- “In case the maximum achievable test concentration is below the limit concentration and evidence is provided that higher testing was not possible with any of the available methods, the highest max attainable conc in accordance with the TG requirements has to be tested”
- “In case the highest (justified) attainable concentration does not yield any mortality, classification is not warranted”

This is in accordance with OECD guideline 39 [19] stating that “if the targeted regulatory limit concentration cannot be achieved by the initial technical procedures, then at least one alternative generation method should be used, ideally using different physical principles but established methodologies”.

A detailed description and justification of the staggered approach including two different methods for aerosol generation was conducted and is described in summary in the Materials and Methods section and the Results section.

In this case, where the maximum achievable test concentration was below the limit concentration of the OECD guideline 436, the highest maximum attainable concentration was tested. For zinc sulphide, the highest attainable concentration did not yield any mortality during the study period. No rat showed clinical symptoms during and upon cessation of exposure. All rats showed a good general health status during the remaining post-exposure observation period of up to 14 days with no indication of a reduction in body functions. In addition, no rat showed gross pathological findings upon necropsy. Thus, an LC50 was not determined in this study, but based on the experimental results, the LC50 value is considered higher than 820.44 mg/m^3^, which is the maximum achievable aerosol concentration resulting in a MMAD in the respirable range of 1–4 µm.

Zinc sulphide is an inorganic substance with very poor water solubility and in vitro bioaccessibility in various biological media. In addition, the substance shows a pronounced tendency to form agglomerates once it becomes airborne. Consequently, the increase in the MMAD results in a decrease in the deposition fraction in the alveolar region of the lungs. According to the IFA (2024) [20], an acute inhalation test (not published; result listed by NIH, USA) was conducted in 1989; the LC50 (rat) value was >5040 mg/m^3^/4 h. This extremely high aerosol concentration suggests that the MMAD value at the time may have exceeded the requested range of 1–4 µm. Nevertheless, this high value indicates the principally low toxic potential of zinc sulphide.

Further toxicological data show a complete lack of local effects towards (mucous) membranes; hence, a very low hazard potential of the inhalable particles is assumed:

- Non-irritating to skin, as assessed in the in vitro skin EpiDerm model and the rabbit skin.
- Non-irritating to the eye, as assessed in the in vitro isolated chicken eye model and the rabbit eye.
- No signs of respiratory irritation, as assessed in the acute inhalation toxicity study.

For more information, please refer to the European Chemicals Agency Zinc Sulphide Registration Dossier [14].

The data presented above in combination with the very low in vitro bioaccessibility in a number of artificial body fluids (artificial sweat, pH 6.5, 24 h: 0.18% Zn release of total Zn; artificial interstitial, pH 7.4, 24 h: 0.025% Zn release of total Zn) and the lack of strong pH effects in a water suspension test (ca. pH = 5.7, Outotec 2010) (for more information, see the European Chemicals Agency Zinc Sulphide Registration Dossier [14]. One may safely assume that even a small fraction of the deposited material does not cause adverse effects in the respiratory tract, as can be assumed for other poorly soluble low-toxicity particles.

The lack of local toxicity is further supported by a 28-day repeated dose toxicity study via oral administration in rats, showing no systemic toxicity or signs of local irritation using gavage treatment. Based on an absence of adverse effects, the NOAEL was set to the limit dose of 1000 mg/kg bw/day. This also underlines the toxicological inertness of zinc sulphide with regard to systemic toxicity (for more information, see the European Chemicals Agency Zinc Sulphide Registration Dossier [14].

## 5. Conclusions

From the results observed in this acute 4 h nose-only inhalation study, the following classification was derived for the test item, i.e., zinc sulphide: Not classified (according to Regulation (EC) No. 1272/2008; EU, 2008) [21]. In conclusion, zinc sulphide is poorly soluble, and the inhalation toxicity is likely attributed to the whole compound rather than the zinc ion with a two-plus charge (Zn^2+^). Based on the research results obtained during dust generation of ZnS, one may conclude that for some substances, inhalation atmospheres cannot be generated at concentrations required by OECD test guidelines. Despite multiple efforts, there appears to be a limitation for some respirable particles such that higher concentrations cannot be achieved. Researchers in the field of inhalation toxicology need to be mindful of this phenomenon. In case no stable atmosphere can be achieved with a test item, it is recommended that these efforts be fully documented to allow an independent reviewer to reproduce all the steps.

## Figures and Tables

**Table 1:** Test Item Data and Properties.

Name Purity Molecular formula Molecular weight CAS number EC name EC number Date of delivery Expiry date	Zinc sulphide >97.3% ZnS 97.46 g/mol 1314-98-3 Zinc sulphide 215-251-3 26 July 2021 26-June-2024 (stable)
Physical aspect Color Odor Density Water solubility	Solid powder, inorganic White Odorless at 20 °C approx. 4 g/cm^3^ <0.0005 g/L (insoluble)

**Table 2:** Study Design (adapted from OECD TG 436).

Test Group	Aerosol Concentration (mg/m^3^) (Presumably)	Treatment	Number of Animals per Conc. (m/f)	Animal Number (DSNN)
Males/Females			Day 14 rec	
1	850	Zinc sulphide	3/3	1101–1103 1201–1203
Total			3/3	

**Table 3:** Aerosol concentrations and mass median aerodynamic diameters (MMADs).

Target Aerosol Concentration (mg/m^3^)	# of Measurements	Approx. 850
**Actual aerosol concentrations** (mg/m^3^)	1	763.77
	2	697.74
	3	1084.34
	4	428.48
**Mean** SD N		743.6 233.3 4
**MMAD** (µm) GSD (-)	1	1.63 2.07
	2	1.37 1.86
**Mean MMAD** (µm) GSD (-) N		1.50 1.97 2

## Data Availability

The datasets used and analyzed during the current study are available from the corresponding author upon reasonable request due to non-disclosure agreement restrictions.

## References

[1] IRIS EPA (2005). Toxicological Review of Zinc and Compounds. https://iris.epa.gov/static/pdfs/0426tr.pdf.

[2] ATSDR (Agency for Toxic Substances and Disease Registry) (2005). Toxicological Profile for Zinc.

[3] REACH Annex VIII: Standard Information Requirements for Substances Manufactured or Imported in Quantities of 10 Tonnes or More. https://echa.europa.eu/regulations/reach/registration/information-requirements.

[4] Russell W.M.S., Burch R.L. (1959). The Principles of Humane Experimental Technique.

[5] Ontox (2021). ONTOX. https://ontox-project.eu/.

[6] (2021). PrecisionTox. https://precisiontox.org/.

[7] Risk-Hunt3r (2021). RISK-HUNT3R. https://www.risk-hunt3r.eu/.

[8] Movia D., Bruni-Favier S., Prina-Mello A. (2020). In vitro Alternatives to Acute Inhalation Toxicity Studies in Animal Models—A Perspective. Front. Bioeng. Biotechnol..

[9] OECD (2009). Test No. 436: Acute Inhalation Toxicity—Acute Toxic Class Method, OECD Guidelines for the Testing of Chemicals, Section 4.

[10] Bruer G.G., Lombaert N., Burzlaff A., Spirlet C., Janssen P., Gödecke P., Ramazanoglu M., Creutzenberg O. (2024). P20-27 Zinc suphide—Considerations and challenges for acute inhalation toxicity testing and classification under REACH. Toxicol. Lett..

[11] Bruer G.G., Ramazanoglu M. Determination of the maximum attainable chamber atmosphere concentration of zinc sulphide—Measurement report, 2022.

[12] Deutsches Institut für Normung e.V. (2006). DIN 55992-1: Determination of a Parameter for the Dust Formation of Pigments and Extenders.

[13] Miller F.J., Asgharian B., Schroeter J.D., Price O. (2016). Improvements and additions to the multiple path particle dosimetry model. J. Aerosol Sci..

[14] European Chemicals Agency Zinc Sulphide: Registration Dossier. https://chem.echa.europa.eu.

[15] OECD (2009). Test No. 403: Acute Inhalation Toxicity, OECD Guidelines for the Testing of Chemicals, Section 4.

[16] OECD (2018). Test No. 433: Acute Inhalation Toxicity: Fixed Concentration Procedure, OECD Guidelines for the Testing of Chemicals, Section 4.

[17] OECD (2024). Test No. 403: Acute Inhalation Toxicity, OECD Guidelines for the Testing of Chemicals, Section 4.

[18] REACH-CLP-Biozid Helpdesk Letter from December 2021.

[19] OECD (2018). Guidance Document on Inhalation Toxicity Studies. OECD Series on Testing and Assessment, No. 39.

[20] IFA (2024). Institute for Occupational Safety and Health of the German Social Accident Insurance. Source: 02071 Toxicological Data, Compiled by the National Institute of Health (NIH), USA, Selected and Distributed by Technical Database Services (TDS), New York, 2009. https://pubchem.ncbi.nlm.nih.gov/compound/14821.

[21] European Parliament and Council (2008). “Regulation (EC) No 1272/2008 on Classification, Labelling and Packaging of Substances and Mixtures (CLP Regulation)”, Official Journal of the European Union. https://eur-lex.europa.eu/eli/reg/2008/1272/oj.

